# Increasing Prevalence, Changes in Diagnostic Criteria, and Nutritional Risk Factors for Autism Spectrum Disorders

**DOI:** 10.1155/2014/514026

**Published:** 2014-02-13

**Authors:** Yasmin H. Neggers

**Affiliations:** Department of Human Nutrition, University of Alabama, P.O. Box 870311, Tuscaloosa, AL 35487, USA

## Abstract

The frequency of autism spectrum disorders (ASD) diagnoses has been increasing for decades, but researchers cannot agree on whether the trend is a result of increased awareness, improved detection, expanding definition, or an actual increase in incidence or a combination of these factors. Though both genetic and multiple environmental risk factors have been studied extensively, many potentially modifiable risk factors including nutritional and immune function related risk factors such as vitamin D, folic acid, and metabolic syndrome have not received sufficient attention. Several recent studies have put forward hypotheses to explain the mechanism of association between both folic acid and vitamin D and autism. A continuous rise in the prevalence of autism in the USA has coincided with a significant enhancement of maternal folate status with FDA mandated folic acid fortification of certain foods starting in 1998. There is also a growing body of research that suggests that vitamin D status either in utero or early in life may be a risk for autism. In this communication, controversies regarding increase in estimate of prevalence, implications of changes in definition, and possible association between some modifiable nutritional risk factors such as folic acid and vitamin D and ASD will be discussed.

## 1. Introduction

Autism, also referred to as autistic spectrum disorder (ASD) and pervasive developmental disorder (PDD), is a frequent and debilitating neurological handicap in children, which is usually diagnosed in early childhood [1]. Since there are no definitive biological markers of autism for a majority of cases, diagnosis depends on a range of behavioral signs. The major symptoms of autism involve problems with communication, social interaction, and repetitive behaviors. Because people with autism can have very different features and symptoms, autism is thought of as a spectrum disorder [2]. The complex nature of these overlapping disorders and changes in clinical definitions over time has resulted in uncertainty in monitoring the prevalence of ASD [2]. Though it is established that autism is a multifactorial condition involving both genetic and a wide range of environmental risk factors, many recently emerging factors such as de novo gene mutation and potentially modifiable nutritional risk factors have not been studied extensively [3–5].

## 2. The Puzzle of Increasing Prevalence

Estimates of the prevalence of the disorder in the USA and other countries are controversial and have been moving towards an apparent increase in rates. Prevalence estimates range from 0.07% to 1.8% [2]. Experts disagree about the causes and significance of the recent increases in prevalence of ASD. Despite hundreds of studies, it is still not known why autism incidence increased rapidly during the 1990s and is still increasing in the 2000s [3].

The importance of accurately identifying children with autism is of utmost importance, particularly given the apparently growing prevalence, considerable family and societal cost, and recognition of the importance of early diagnosis and intervention. In 1990, the Congress added autism as a separate category to federal law that guarantees special education services [6]. Since then there has been an explosion of autism related treatments and services.

Though it is difficult to discuss the prevalence of autism without a universally acceptable definition, for this paper, unless otherwise indicated, Diagnostic and Statistical Manual of Psychiatric Disorders IV-TR (DSM-IV TR) (the American Psychiatric Association (APA), 2000) criteria which recognizes the category of Pervasive Developmental Disorders, under which the diagnoses of Autism, Asperger syndrome, and Pervasive Developmental Disorder Not Otherwise Specified (PPD-NOS) fall (along with Rett syndrome and Childhood Disintegrative Disorder), will be used [7, 8]. Later in the paper a comparison of the existing DSM-IV and newly instituted DSM-5 criteria will be discussed. With the next iteration of the DSM, the DSM-5, the category of Autism Spectrum Disorder (ASD) is officially recognized, having previously been used to capture autism, Asperger syndrome, and PDD-NOS and continuum of impairments represented by these diagnoses [9].

Recently (March 30, 2012), data from the Autism and Developmental Disability Monitoring Network Surveillance (ADDM), reported by the Centers of Disease Control and Prevention (CDC), indicated a significant increase in prevalence of ASD [10] as follows.

- For 2008, the overall estimated prevalence of ASD among 14 ADDM sites was 11.3/1000 (1 in 88) children aged 8 years. Age varied by subtype of ASD (median earliest age ASDs were documented in their record: 4 years, 6 month).
- This estimate varied widely across all sites, from 1 in 210 in Alabama to 1 in 7 in Utah (range: 4.8–21.2/1,000 children).
- There were wide variations by gender and racial/ethnic groups.
- Approximately 1 in 54 boys and 1 in 252 girls were identified as having ADS.

Race/ethnicity:

- white, non-Hispanic: 12.0/1,000,
- black, non-Hispanic: 12.2/1,000,
- Hispanic: 7.9/1,000,
- Asian or Pacific Islander: 9.7/1,000.

The new numbers of ASD cases reported in 2012 by the CDC are the latest in a series of studies that have steadily raised the official autism estimates. These new figures mean that autism is nearly twice as common as estimated only five years ago. If these estimates for ASD (1 in 88 children) are valid and not an artifact of confounding or systematic bias due to better screening and ascertainment, then ASD affects more than 1 million children and adolescents in the United States.

A Comparison of these 2008 findings with earlier surveillance years indicates a dramatic increase in ASD prevalence over a short period:

- an estimated increase of 78% when the 2008 data was compared with data from 2002 (11.0/1,000 in 2008 versus 6.4/1,000 in 2002),
- an estimated increase of 30% when data from 2008 was compared to 2004 (11.0/1,000 in 2008 versus 8.0/1000 in 2004),
- there was an estimated increase in prevalence of 23% when the data from 2008 was compared with data from 2006 (11/1000 in 2008 versus 9/1000 in 2006).

*Eight-Year-Old Children Diagnosed with Autism 2002–08*

**Table:** 

2002	1/150	0.66%
2004	1/125	0.80%
2006	1/110	0.90%
2008	1/88	1.14%

Because the ADDM Network sites do not make up a nationally representative sample, these combined prevalence estimates cannot be generalized to the USA as a whole.

Researchers have suggested that ASD onset, and prevalence are similar across European and North American populations [11]. In Australia, a population based study indicated that the prevalence of ASD increased by 11.9% per annum among children born between 1983 and 1999 and diagnosed by age 8. With exception of Japan, the data are insufficient to measure ASD prevalence accurately in other cultures [12]. For most studies conducted since 2000 in different geographical regions and by different investigators, estimates converge to a median of 17/10,000 for AD and 62/100,000 for all PDDs. A modified version of summary of prevalence estimates of AD and PDD in various regions of the world since 2000 provided by Elsabbagh et al. [13] is presented in Table 1. For most regions median estimates are recorded except when there were too few estimates available within a given region. It should be noted that these values represent the best estimates currently available and represent median figures with substantial variability across the studies.

## 3. Possible Reasons for Increasing Prevalence

With steady increase in prevalence of ASD over the last decades, the question arises about how much of this increase is real and how much of it is due to wider screening, broadening ASD diagnostic criteria, lower age of at diagnosis and intervention, a greater public awareness, and parental advocacy. Increase and discrepancies in prevalence estimates of ADS may be partly explained because most studies have focused on populations that are more likely to include children with ASD, that is, those with histories of special needs or developmental delays. In a few studies which have included children with mainstream education, participation rate and sample size have not been adequate. The first population based autism prevalence study in Korea with 55,266 children between 7–12 years of age reported a striking difference in the prevalence of ASD of 2.64% as compared to previously reported estimates ranging from 0.6 to 1.8% [11]. In this study 2/3 of ASD cases in the overall sample were mainstream school population, undiagnosed and untreated.

Children with such diagnosis often receive state financed support services, which some experts believe may have contributed to an increase in prevalence.

### 3.1. Diagnosis Criteria

Among the most common issues cited for increase in ASD prevalence are the frequently changing diagnostic criteria for the ASD and even the definition of subcategories of autism or autism related disorders [5]. Formal diagnostic criteria did not emerge until the publication of the third edition of Diagnostic and Statistical Manual of Mental Disorders (DSM-III; American Psychiatric Association (APA)) in 1980. Thus, diagnoses made prior to this publication were not evaluated according to the same, if any, set criteria. The ASD criteria were then modified and expanded throughout the successive versions of DSM (APA, 1987, 1994, 2000 (DSM IV-TR)) up until its latest revision, the DSM-5, May 2013, in which the definition of autism has been changed [14]. A number of changes occurred with each updated version of the DSM, including age of onset criteria, what diagnoses were classified as ASD, symptom inclusion, and the number of symptoms needed for diagnosis [14, 15]. In response to the debate over the role of diagnostic substitution in increasing prevalence of autism, King and Bearman estimated the extent to which the increased prevalence of autism in California can be explained by changes in diagnostic practices [16, 17]. The odds of a patient receiving an autism diagnosis significantly increased in periods in which the practices for diagnoses changed. Between 1992 and 2005 the estimated increase in prevalence of autism was reported to be 26.8%, 95% C.I. = 16.27–36.48.

As discussed later in detail, among other modifications, the latest version (DSM-5, 2013) includes a new “autism spectrum” category describing symptoms that usually appear before age 3 and encompasses children with “autistic disorder” now referred to as severe cases, plus those with two high functioning variations [18]. Furthermore, another frequently used classification system, the International Classification of Diseases Series, has separate diagnostic criteria from most of the DSMs [19, 20]. However, a more consistent diagnosis world-wide may have resulted because the criteria put forward in the DSM-IV (APA 1994) and DSM-IV-TR (APA, 2000) were more comparable to those set by the ICD-10 (WHO, 1992 [20]). Given all the modification and expansion of categories, it is not surprising that definitions of ASD may have a part in the increase in the rising prevalence rates.

### 3.2. Inaccurate Diagnosis

Among the common factors cited for the increase in ASD some can be linked to inaccurate diagnosis [14]. Clinically derived diagnoses are often made following brief observation using DSM or ICD guidelines but not systematic testing. When tests are used, they can vary widely in the type of tests used as well as in who the respondents are [21, 22]. Also, a range of clinicians including clinical psychologists, school psychologists, psychiatrists, and neurologists may evaluate the condition. The amount of training and experience they have with ASD varies widely. On the other hand, those using research criteria are more likely to employ homogenous protocols with a standard test battery conducted by professionals with specific training in ASD [21]. As mentioned before, in the current climate, a child with ASD and intellectual disability would be more likely to receive ASD versus intellectual disability diagnosis in the past [23].

### 3.3. Environmental Components

Major improvements in perinatal and neonatal care have resulted in greater survival rates for premature infants. These premature and/or very low birth weight babies are likely to be affected by neurodevelopmental disorders, including autism, at a much higher rate than the general population [24] Prenatal risk factors and high rate of neonatal hospitalization have also been suggested as a possible cause for increased prevalence of autism.

### 3.4. Research Methodology

Prevalence estimates for ASD and related developmental disorders are derived from a variety of ways including reports from registries, retrospective reporting, telephone interviews, and whole area surveys [25, 26]. These differences among methods of ascertainment and research methodologies would inevitably affect prevalence estimates. In most recent study conducted by the CDC in 2009, different records were available dependent on individuals being assessed with some only having medical records available while others also had accessible education records. This resulted in higher prevalence rates in the regions with access to both medical and educational records [14]. Thus, it is obvious that rates would differ for similar reasons across studies if such differences resulted in the same study. Also, when very young children are involved, it is important to consider autistic regression. While working with prevalence data on children under the age of 3 years, it is likely that actual numbers may be higher later in childhood [27].

### 3.5. Cultural Factors and Awareness

Investigators have noted that prevalence of ASD has risen dramatically over last decade and a half and most increase in rates has been reported in the developed countries [12, 28]. These studies pointed out that Israeli born children showed higher rates of ASD than Ethiopian children. Furthermore, Israeli children of Ethiopian extraction showed rates of ASD at a much higher rate than the children born in Ethiopia. Therefore, acculturation and other social factors seem to play a role in the ascertainment of children with ASD and thus overall reported rates of ASD. Another relatively recent important phenomenon which may have resulted in increased reporting of ASD cases is discussions of ASD and problems with services related to treatment and diagnoses in the media. Celebrities making ASD awareness a cause due to an acquaintance or they themselves having an affected child may have led to increased reporting.

ASD is a relatively new concept in most Eastern and Middle Eastern countries [29, 30]. Also, many technically advanced countries like China and India have not yet developed proper screening instruments to accurately enumerate the prevalence of autism related disorders [31–33].

Though there is valid evidence that current ASD prevalence estimates are significantly higher than those that were reported in previous decades and a portion of this documented increase may be attributed to a true increase in prevalence, it is quite plausible that some of this increase in prevalence rate is due to bias and confounding related to factors mentioned earlier. So it is not just a coincidence that using different diagnostic criteria and other related factors provides researchers with varying rates of prevalence of ASD.

## 4. Latest Changes in ASD Definition (DSM-5)

The Fifth Edition of the Diagnostic and Statistical Manual of Mental Disorders (DSM-5) was released at the American Psychiatric Association's Annual Meeting in May 2013; it marked the end of more than a decade's journey in revising the criteria for the diagnosis and classification of mental disorders (http://www.dsm5.org). For the first time in nearly two decades, a panel of American Psychiatric Association researchers has rewritten the definition of autism. The group's board of trustees voted on the proposals in December 2012 [34]. Field trials have been conducted for validation of the DSM-5 criteria for ASD. These trials were conducted to evaluate new diagnostic criteria that contain several important modifications relative to DSM-IV-TR diagnoses into a single broad ASD [35].

### 4.1. Among the Major Changes

- A new “autism spectrum” category was created describing symptoms that usually appear before age 3 and would encompass children with “autistic disorder” now referred to as severe cases, plus those with two high functioning variations.
- Autistic disorder and high functioning variations: Asperger's disorder and PDD-NOS would be eliminated, but their symptoms will be covered under a new category.
- Another new category “social communication disorder” would include children who relate poorly to others and have trouble reading facial expressions and body language.

Table 2 presents diagnoses according to DSM-IV-TR and patient records and DSM-5 draft along with suggested modified DSM-5 draft [18]. Frazier et al. [6] evaluate the validity of the DSM-5 criteria for ASD by analyzing symptoms of 14,744 siblings (8,911 ASD and 5, 863 controls) reported in a national registry, the Interactive Autism Network. Results of this study supported the validity of proposed DSM-5 criteria for ASD as provided in Phase I Field Trials criteria. Frazier et al. [6] concluded that increased specificity of DSM-5 relative to DSM-IV-TR may reduce false positive diagnoses, a particularly relevant issue for low base rate in clinical setting. Sensitivity and specificity of DSM-IV-TR, DSM-5, and modified DSM-5 criteria in Table 3 show substantial but not complete, overlap (*k* = 0.74, agreement = 87.1 %). DSM-5 criteria have superior specificity but lower sensitivity relative to DSM-IV criteria (*P* < 0.001). According to this comparison, DSM-5 could reduce false positives by more than four times the estimated DSM-IV-TR rate. Despite some limitations, the findings of this study provided a comprehensive validation of DSM-5 criteria and provided promising leads for future examinations of DSM-5 algorithms. Relaxed DSM-5 criteria may further improve identification of ASD and decrease societal costs through appropriate early diagnosis and maximizing limited intervention resources.

Although DSM-5 is now complete, a great deal of work, including the proper use of DSM-5, including providing training materials, questions about its implementation in clinical care, and research; clarifying concerns about the new ICD codes and insurance billing; and correcting any errors remains to be done [34, 36].

There is concern among several groups of autism advocates and parents that the proposal will exclude as many as 40% of children now considered autistic. But the members of the panel that proposed changes maintain that none of the affected children will be left out and that the revision is needed to remove confusing labels and clarify that autism can involve a range of symptoms from mild to severe. The DSM-5 Neurodevelopmental Disorders Workgroup of The American Psychiatric Association [35, 37] has provided several documents and webinars to explain the changes and strengths of DSM-5, including the following statement in a symposium by Dr. W. D. Kaufman [36, 38].

### 4.2. Criticisms of DSM-5 ASD Criteria

“Changes in criteria threaten services delivery.”

- Actually, a single diagnosis of ASD will improve access to services.
- PDD-NOS and Asperger disorder do not qualify for services in 14 states.
- Apparent biases in diagnostic labeling with rich, white males receiving (less-stigmatizing) dx of Asperger disorder, while poor, nonwhite males, and all females receive PDD-NOS (or autism).

It is somewhat ironic that these changes in the definition of autism are being proposed in the same year when CDC has reported a significant increase in autism cases (1 in 88 children as compared to previous estimate of 1 in 110) and advocacy groups have seized on the new increased rate as further evidence that autism research and services should get more attention.

## 5. Emerging Nutritional Risk Factors for Autism

In spite of decades of extensive research, the etiology of ASD is unknown. A number of risk factors being investigated include genetic, infectious, metabolic, nutritional, and environmental factors, with specific causes known in less than 10 to 12% of cases [39]. In most cases, specific underlying causes cannot be identified. ASD is believed to have genetic and environmental origins, yet only in a small fraction of individuals specific causes can be identified. Though it is well established that ASD has a significant genetic component; for at least 70% of cases the underlying genetic cause is unknown [36, 40]. In this commentary a few nutrition related emerging risk factors which encompass both genetic and environmental aspects will be discussed.

Though ASD is considered an autoimmune disease [39, 41] it also appears to have several important diet and nutrient related risk factors. Although genetic contributions to autism etiology are well accepted, the rising prevalence and inconsistent finding from genetic studies suggest a role for interactions between susceptibility genes and environmental factors [40, 42]. A growing body of literature suggests that certain modifiable risk factors such as maternal metabolic syndrome and certain vitamins such as vitamin D and folic acid either in utero or early life, may be associated with increased risk of autism [43–45].

## 6. Association between Folic Acid and Autism

Folic acid, the oxidized form of the vitamin used in fortified foods and in supplements and folate, the reduced form of the vitamin found in foods, is essential for cell replication during periods of growth such as pregnancy, particularly during early prenatal periods [46]. Supplementation with folic acid during the time of conception (perinatal period) reduces the risk of neural tube defects in offspring [46, 47]. This protective effect *led to mandatory fortification of flour and cereal products in several countries *[46, 48]. Also, there is evidence that maternal folic acid supplementation during pregnancy may reduce the risk of other neurodevelopmental disorders in children. Involvement of alteration in the folate methionine cycle may play a key role in the etiology of autism [49–52]. The role of nutritional factors was suggested in a study reporting association between autism and short intervals between pregnancies. A leading hypothesis put forward is that low folate status (resulting from maternal nutritional depletion) is associated with increased risk of autism [53]. Folate, a water soluble B vitamin, plays several key roles in maintenance and repair of the genome, regulation of gene expression, amino acid metabolism, and the formation of myelin. Autism is a neurodevelopmental disorder that potentially originates during early pregnancy when folate is known to be critical [54–58]. Thus, the observation that increased odds of autism in the second born sibling in pregnancies after a less than 1 year interval is consistent with the hypothesis that folate status might be related to autism risk [53, 56, 59].

## 7. Folic Acid: ASD Hypotheses

Folate and folic acids (used in supplements and food fortification) are sources of single-carbon units essential for cell replication, including DNA replication and DNA, RNA, and protein methylation. Folates are also critical in de novo methionine generation [56]. Given the interest in prenatal risk factors for ASD, numerous researchers have hypothesized that perinatal nutritional exposures, such as maternal folic acid intake, may be associated with increased risk of autism. This is biologically plausible, since autism is a neurodevelopmental disorder that potentially originates during early pregnancy when folate is known to be critical [57]. Periconceptional folic acid supplementation in randomized clinical trials has reduced the risk of neural tube defects up to 70% and has resulted in fortification of cereal products in the USA and the recommendation that all women of child bearing age consume 400 µg/day of folic acid [58].

Several investigators have put forward hypotheses to explain the mechanism of association between folic acid and autism [59–63]. Although genetic contributions to etiology are widely accepted, because of increasing temporal trends in autism prevalence, wide variations in the clinical presentation between family members and among affected individuals, inconsistent findings from gene studies, and less than 100% concordance in monozygotic twins have led to suggestions of a role for multiple interactions between susceptibility genes and environmental factors [60]. Ramaekers et al. [62] identified reduced 5-methylenetetrahydrofolate (5MTHF) transport into the cerebrospinal fluid (CSF) in two autism spectrum disorders, that is, Rett syndrome and infantile low-functioning autism due to folate receptor autoimmunity. In spite of normal serum folate, CSF 5MTHF was low in 23 of 25 patients and was explained by serum folate receptor (FR) autoantibodies blocking the folate binding site of the membrane attached FR on the choroid epithelial cell. A partial or complete clinical recovery was reported after 12 months of oral folinic acid supplements in affected children [62]. These researchers suggested that FR autoimmunity and cerebral folate deficiency appear to play a crucial role in the pathogenesis of autism spectrum disorders or in a particular subgroup of the autism spectrum.

Other hypotheses have suggested that the association between folate and autism may be due to reduced activity of polymorphic forms of some key enzymes required for folate metabolism. It is well established that there has been a significant enhancement of maternal folate status since FDA mandated folic acid fortification of certain foods (1998) which has resulted in decreased incidence of NTDs during mid-2000s [48]. This same time period coincides with the apparent beginning and continuous rise in the prevalence of autism and related disorders in the USA Investigators have wondered whether these similar time frames of change in maternal folate status and possible autism prevalence are a random event or that the improved maternal and resulting fetal folate status has played a role [60]. Rogers and other researchers [60, 63–65] have explored the possibility that a particular polymorphic form of a key enzyme methylenetetrahydrofolate reductase (MTHFR), required for activation of folate for methylation in neurodevelopment, exhibits reduced activity under low or normal folate levels but normal activity under higher folate nutritional status. In several studies, higher plasma homocysteine levels than noncarriers resulting from the presence of polymorphic forms of MTHFR during reduced or normal folate status have been shown to result in increased rates of miscarriages via thrombotic effects [66, 67]. However, under the condition of enhanced folate status during the perinatal period, the incidence of hyperhomocysteinemia is reduced and thereby masks the latent adverse effects of the presence of this polymorphic form of MTHFR during pregnancy [68]. This polymorphism, although common in the normal population, is found in significantly higher frequency in children with autism. Thus it is hypothesized that enhanced folate status during pregnancy from fortification could have increased the survival rate of fetuses with genetic polymorphism such as MTHFR 667 C > T, which are associated with high homocysteine and subsequently require higher amounts of folate for normal methylation needed for proper neurodevelopment [69]. Such polymorphisms have been observed in higher frequencies in children with autism, suggesting that these children might be genetically predisposed to less efficient folate metabolism and function [62, 70]. 

Several epidemiological studies have tested the above mentioned hypotheses regarding the association between folic acid/folate and ASD. In this section studies are reported in chronological order and by study design. Relatively recent studies (starting in 2000) with well-designed methodology are included to examine the evidence for the involvement of folic acid/folate or alteration in folate metabolism and risk of ASD. A summary of these studies is presented in Table 4. With the exception of a prospective study in a Norwegian children cohort by Suren et al., [53] most of these investigations consist of case control design, where maternal perinatal folate or multivitamin supplementation in children with ASD was retrospectively compared with maternal perinatal folate or multivitamins supplement use of healthy children. Schmidt et al. [61] evaluated the association between autism and the use of perinatal vitamins and one carbon metabolism gene variant. A few investigators have also evaluated the effects of folate metabolites and their possible role as oxidative stressors as a risk factor for autism and the effect of interaction between maternal folate status and maternal genotype and risk of autism [58, 61]. Results of these studies indicate an association between maternal perinatal folate status and ASD. Significant interaction effects have been reported for maternal MTHFR 677 TT, CBS rs234715 GT +TT, and child COMT 472 AA genotype, with greater risk for autism when mothers did not report taking prenatal vitamins periconceptionally. Schmidt et al. [61] have observed greater risk for children whose mothers had other one-carbon metabolism pathway gene variants and no maternal vitamin intake. As stated earlier, most of these investigations were retrospective case control studies with possibilities of various types of biases including differential misclassification of disease or/and exposure. Currently several randomized clinical trials are underway to clarify and confirm the association between periconceptional folic acid intake and autism [71].

## 8. Vitamin D Status as a Risk Factor for Autism

Vitamin D, a secosteroid, is structurally part of a group of sterols with a crucial role in calcium and phosphorous metabolism [72]. The main source of vitamin D is the solar ultraviolet B radiation related conversion of 7-dehydrocholesterol to previtamin D3 in the skin and a lesser amount of vitamin D from food [73, 74]. There is a growing body of research that suggests that vitamin D status either in utero or early in life may be a risk of autism. Autism is considered an autoimmune disease and appears to have important risk factors in utero as indicated by a highly increased frequency of congenital malformations [75–78]. Also, several studies have reported seasonality to be associated with excess births of autistic infants, with March as a peak birth month in several countries including Boston, Denmark and Sweden [79–86].

Grant and Soles [72] used epidemiological data for seasonal variation of birth rates and prevalence of infantile autism disease (IAD) to examine whether maternal vitamin D deficiency was a risk factor for IAD for cohorts born before 1985. Data from seven studies reported spring to summer excess birth rates for IAD, the season progressed from close to 30°N latitude, spring/summer in midlatitudes, to the highest latitude in winter.

## 9. Vitamin D and Autism Hypotheses

Several hypotheses have been proposed and tested to explain the association between vitamin D status of mother and/or autistic child. Investigators have advanced the hypothesis that association between vitamin D and autism could be explained by the relationship between seasonality and low maternal serum 25-hydroxyvitamin D concentration (a valid indicator of vitamin D status) [73]. Because activated vitamin D (1, 25-dihydroxy vitamin D), a secosteroid, upregulates the DNA repair gene, vitamin deficiency during development may inhibit the repair of de novo DNA mutations in fetuses and infants and thus contribute to risk of autism.

Other mechanisms which may explain the effect of vitamin D on autism symptoms are that vitamin D may reduce the severity of autism through its anti-inflammatory actions, increasing T-regulatory cells, antiautoimmune effects, and upregulating glutathione, a scavenger of oxidative byproducts [81–84]. Active vitamin D (1, 25-dihydroxy vitamin D), like other hormones, acts as a molecular switch, activating many target genes via the vitamin D receptor (VDR). Cannell proposed a theory for the genetic component of autism involving the vitamin D system [84]. Cannall used one of the inherited components of the vitamin D system and vitamin D related disease, atherosclerosis, as a model to illustrate the mechanism of association between autism and the vitamin D system. Schnatz et al. in a study to evaluate whether heritable variations in the expression of VDR in atherosclerosis was linked with the severity of the disease showed that 1,000 I.U. of vitamin D_3_/day supplement reduced the formation of atherosclerotic plaques in the arteries of monkeys [85]. A strong negative association (*P* < 0.001) was found between inherited VDRs and severity of atherosclerosis. Similarly, significant changes in composite health outcomes (cancer, myocardial infarction, and hip fractures) occurred with respect to a combination of serum 25-OH vitamin D levels and VDR alleles. Low serum levels of 25-hydroxy D and two minor alleles were associated with increased risk (hazard ratio = 1.82, 95% C.I. = 1.31−2.54). Grant and Cannell proposed that a similar mechanism may describe autism. Heritability of autism may be explained by quantitative genetic variations in some facet of vitamin D metabolism, such as VDR or in the enzymes that activate vitamin D [86]. Lower levels of 25-hydroxy vitamin D have been reported by several investigators. A study conducted in Saudi Arabia found that despite the same amount of sun exposure, not only was there much lower levels of 25-hydroxy vitamin D in autistic children as compared to controls (about half of those of controls), but also there was an extremely strong negative correlation coefficient (*R* = −0.84) between vitamin D levels and autism severity on the autism severity scale [75]. These observations imply that vitamin D levels in autistic children are highly heritable [87]. However, recent studies have indicated that a genetic disease may not be inherited [88, 89]. De novo genetic mutations, such as those seen in autism and schizophrenia may occur during the lifetime and may not be inherited. These observations may explain the genetic findings of autism, since the most common genetic finding is multiple small genetic de novo mutations. Some studies have indicated that most of the observed de novo point mutations are not related to the etiology of autism [90]. A few de novo mutations are associated with increased risk but are distributed across many chromosomes and are not severe enough to explain the disease [91, 92]. This implies that genetic defects in autism are often minor compared with well recognized defects such as those which occur in trisomy 21. However, multiple studies have confirmed the contribution of rare de novo copy number variations to the risk of ASD [93, 94]. Sanders et al. have indicated that de novo mutations are strongly associated with autism. Using whole-exome sequencing of 928 individuals, including 200 phenotypically discordant sibling pairs, has shown that highly disruptive de novo mutations in brain-expressed genes are associated with ASD and carry large effects [89].

Another proposed hypothesis for the role of vitamin D in development of autism has to do with Vitamin D's function in the DNA repair and maintenance [95]. Dihydroxy D_3_ protects cells by upregulating a DNA repair gene that helps to repair double-strand breaks. Vitamin D may guard the genome via control of DNA repair [96, 97]. In a randomized controlled trial in humans, 800 IU of vitamin D increased the levels of Bax, which helps to stop mutations by promoting apoptosis [98]. Thus, it is possible that widespread point mutation and de novo DNA mutation damage seen in autism could be an effect, not a cause. The effect of a genetically impaired vitamin D system combined with inadequate amounts of vitamin D may be associated with de novo mutations reported in autism.

The search for a genetic basis of autism is an ongoing complicated process. The quantitative genetic variation in various components of vitamin D, as proposed by Cannall, may be a piece of this puzzle. The role or roles vitamin D may play in development of ASD may be resolved by randomized clinical trials of prenatal vitamin D supplementation or vitamin D supplementation of newborns or in early childhood. Several clinical trials like “Vitamin D to prevent autism in newborn siblings” are currently being conducted (http://clinicaltrials.gov/ct/show/NCT01366885?Term=Vitamin+D+and+autism) [99, 100].

## 10. Maternal Metabolic Conditions, Prenatal Inflammation, and Risk of Autism

Gestational diabetes is known to be associated with genetic development impairments in offspring. Also, diabetes is more common in autistic children [101, 102]. However, research regarding specific associations between autism and maternal diabetes has produced inconsistent results. Few studies have examined related conditions accompanied by underlying increased insulin resistance and their relation with developmental disorders [103, 104]. In a well-designed population based case control study, Krakowiak et al. evaluated whether diabetes, hypertension, and obesity also referred to as metabolic conditions (MC) during pregnancy are associated with ASD and developmental delays (DD) in the offspring [101]. Subjects were 2–5 years old children enrolled in the previously mentioned CHARGE study with confirmed ASD diagnosis. Information regarding maternal condition was ascertained from the medical records or a structured interview with the mother. All metabolic conditions were more prevalent in case mothers were compared with controls. These conditions, collectively, were associated with increased risk of ASD and DD as compared to controls (odds ratio = 1.61, 95% CI = 1.10−2.37 and 2.35, 95% CI = 1,43−3.88 resp.). Mitri et al. in a double blind randomized trial showed that 2,000 IU/day of vitamin D3 in adults decreased insulin resistance [102]. In a study of nonautistic children with 25-hydroxy D levels below 10 ng/mL (poor vitamin D status), insulin resistance was 7 times higher than that in children with vitamin D levels above 30 ng/mL [103]. These studies indicate that maternal MCs, particularly diabetes, may be associated with neurodevelopmental problems in children and vitamin D supplementation early in childhood should be given further consideration in treatment of ASD [103–105].

Another emerging risk factor associated with the prenatal environment being examined as a possible risk factor for ASD is prenatal inflammation. Few investigators have reported that elevated plasma cytokines and maternal C-reactive protein are associated with ASD [106, 107]. To test a potential role for immune dysfunction in ASD, differential cytokine release in plasma of 2–5-year-old children with ASD was compared with age matched typically developing (TD) children and children with developmental disabilities (DD). This population based cohort was a part of the CHARGE study [106]. A significant increase in plasma cytokines in the ASD group compared to the TD controls (*P* < 0.04) was observed. It was noted that the increased cytokine levels were predominantly in children with a regressive form of ASD. Thus, a significant shift in cytokines profiles was evident. The investigators concluded that ongoing inflammatory response may be linked to disturbance in behavior in ASD. In a similar vein, Brown et al. investigated whether inflammation during pregnancy may represent a pathway by which infections and other insults during prenatal period may increase the risk for ASD [107]. Brown et al. [107] conducted a prospective study with nearly complete ascertainment of pregnancy, early gestational C-reactive protein (CRP). An established biomarker of inflammation was measured in maternal serum of a very large cohort (*n* = 1.2 million) in Finland. Increasing maternal CRP levels, analyzed as a continuous independent variable, were significantly associated with autism in offspring. This acute phase reactant protein is synthesized by hepatocytes in response to IL-6, though other cytokines, including interleukin-1*β* and tumor necrosis factors, also have roles in CRP induction [107–110]. IL-6 was elevated in the cerebellum of autistic subjects which is consistent with maternal immune activation in exposed offspring. These findings may have important implications for prevention, because many standard approaches exist to decrease the incidence of infection. 

## 11. Conclusions

Though more than $1 billion has been spent during the past decade on ASD research, it is disappointing that the estimated prevalence of ASD has increased by 78% as compared to the 2002 estimates. Also, in the past two decades not much progress has been reported in untangling risk factors associated with ASD, or the factors which may be related to increased prevalence of ASD. However, recent identification of de novo gene mutation may account for a small but significant percentage of ASD cases. This research has opened up a large field for future discovery, diagnostics, and hopefully therapeutics. Furthermore, other modifiable, nutrition related risk factors such as folic acid, vitamin D, and maternal metabolic syndrome may account for some proportion of increase in ASD prevalence. Preponderance of evidence suggests a linkage between poor maternal folic acid status and/or folic acid levels during early childhood and autism related disorders. A better understanding of the metabolic basis of autism seems to have potential for the development of laboratory-based testing for autism diagnosis. Although findings so far do not conclusively implicate a dysfunctional folate-methionine pathway in the etiology of autism, the topic obviously deserves more effort and scrutiny studies have indicated that folic acid supplementation can normalize serum folate and folate-methionine metabolites. Whether this normalization affects behavioral measures is yet to be determined.

More research is also needed to further investigate quantitative genetic variations in various components of the vitamin D system. Poor maternal vitamin D status or early childhood vitamin deficiency or low activity of various vitamin D related enzymes may result in deficient activity in the vitamin D system crucial for brain development. Any of these conditions may result in ASD related disorders. Plausible mechanisms have been put forward to explain the palliative role of vitamin D in children with autism via DNA repair, anti-inflammatory actions, autoimmune activities, and increase in regulatory T cells or stimulation antioxidant pathways. Recruitment for an open label clinical trial to evaluate large doses of vitamin D in autistic children as a new treatment modality, with target 25-hydroxyvitamin D3 levels of 80 ng/mL is underway [99].

It is obvious that ASD research has a long way to go before developing precise yet inclusive diagnostic criteria, pinpointing relevant risk factors, and providing valid reasons for increasing prevalence of ASD.

## Figures and Tables

**Table 1:** Summary of prevalence estimates of AD and PDD across the world regions since the year 2000*.

Region	AD estimates			PDD estimates		
	Median	Range	Number of estimates	Median	Range	Number of estimates
Europe	19	7–39	16	62	30–116	14
America	22	11–40	7	65	13–110	12
Western Pacific	12	2.8–94	12	—	—	3
Southeast Asia	—	—	1	—	—	1
Eastern Mediterranean	—	—	0	—	—	3
Africa	—	—	0	—	—	0
All	**17**	**2.8**–**94**	**36**	**62**	**1**–**189**	**33**

AD: autistic disorder.

PDD: developmental disorder.

*Adapted from Elsabbagh et al., 2012 [13].

**Table 2:** Diagnoses according to DSM-IV-TR and patient records, DSM-5 draft, and suggested modified DSM-5 draft*.

Diagnosis	DSM-IV-TR and patient records				DSM-5 draft^a^		Suggested modification^b^
	Prevalence/1,000	95% CI	*n* (%)		*n* (% of DSM-IV-TR)		*n* (% of DSM-IV-TR)
ASDs^c^ (all)	8.4	6.1–11.5	37 (100)				
ASDs (≥70)^d,e^	0.45	0.1–1.6	2 (5)				
ASDs (≥50)^f^			26	→	12 (46%)	→	25 (96%)
ASDs (≥70)^f,e^	5.0	3.3–7.5	22 (60)	→	8 (36%)	→	21 (95%)
ASDs (50–69)^f^	0.9	0.4–2.3	4 (11)	→	4 (100%)	→	4 (100%)
ASDs (35–49)^g^	1.6	0.8–3.3	7 (19)				
ASDs (20–34)^g^	0.45	0.1–1.6	2 (5)				
ASDs (<20)^g^			0 (0)				
Autism (all)	4.1	2.6–6.4	18 (48.5)				
Autism (≥70)^f^	2.5	1.4–4.4	11 (61)	→	8 (73%)	→	11 (100%)
Autism (50–69)^f^	0.9	0.4–2.3	4 (22)	→	4 (100%)	→	4 (100%)
Autism (35–49)^g^	0.7	0.2–2.0	3 (17)				
Autism (20–34)^g^			0 (0)				
Autism (<20)^g^			0 (0)				

AS^f^	2.5	1.4–4.4	11 (30)	→	0 (0%)	→	10 (91%)

The rest^d,g^	1.8	0.9–3.6	8 (21.5)				

^a^Evaluated in 82 participants (full-scale intelligence quotient (FSIQ) ≥50).

^b^Mattila et al.'s modification of DSM-5 draft criteria [18].

^c^Including autism, Asperger's syndrome (AS), and “the rest.”

^d^According to parents' developmental questionnaire and patient records (one with AS and one with AS traits).

^e^Of all autism spectrum disorders (ASDs), 65% high-functioning.

^f^Based on screening and examinations in the epidemiological study.

^g^Drawn from patient records (three with autism, five with autistic traits, and one with pervasive developmental disorder).

*Adapted from Mattila et al., 2011 [18].

**Table 3:** Sensitivity and specificity of DSM-IV-TR, proposed DSM-5, and modified criteria for clinical diagnosis and empirically derived classifications*.

	Clinical ASD diagnosis		Empirically derived classifications	
	Sensitivity	Specificity	Sensitivity	Specificity
DSM-IV-TR	0.95	0.86	0.92	0.83
DSM-5: field trial phase I	0.81	0.97	0.78	0.95
DSM-5: relaxed algorithm	0.93	0.95	0.89	0.92
DSM-5: without high-functioning symptoms	0.64	0.98	0.61	0.97
DSM-5: without sensory sensitivity/interests	0.78	0.97	0.75	0.96
DSM-5: one symptom per criterion	0.96	0.90	0.92	0.87

Comparison of DSM-IV-TR and proposed DSM-5 criteria was done using a subsample with complete data on the Social Communication Questionnaire (SCQ) and Social Responsiveness Scale (SRS) (*N* = 6,426).

*Adapted from Frazier et al. [6].

**Table 4:** Association between folate/folate metabolites, gene polymorphism in folate and Autism*.

Authors, country, and year of publication	Study design and subjects	Case definition	Outcome measure	Results
Boris et al. USA, 2004 [69]	**Case control study** *Cases:* 168 Caucasian children 148 (84.5%) males 26 (5.5%) females *Controls:* 5389 Caucasians	73.8% autism, 26.3% PDD diagnosed by neurologist, psychiatrist, or neuropsychologist. DSM IV criteria used for diagnosis	Frequency of MTHFR alleles 677C→T 1298A→C in cases and controls	Significantly increased (*P* < 0.0001) frequencies of homozygous mutation 677CT allele (23% in cases versus 11% in controls) and heterozygous 677CT allele (56% in cases versus 41% in controls). Overall increased risk of ASD associated with common mutations affecting the folate/methylation cycle.

James et al. USA 2006 [63]	**Case control study** *Cases:* 80 Caucasian children from autism clinics 89% males 11% females Mean age 7.3 ± 3.2 yrs. *Controls:* 73 unrelated healthy Caucasian from a metabolic study Mean age: 10.8 ± 4.1 yrs.	Autism diagnoses: by independent specialists DSM IV or ADOS or CARS criteria	Plasma levels of folate metabolites: Methionine, Homocysteine Cystathionine, Cysteine SAM, SAH Glutathione Allele frequency: RFC 80>A TCN2 776G>C COMT 472G>A MTHFR 667>T	Plasma methionine and SAM/SAH ratio was significantly lower in autistic cases as compared to age matched controls (an indicator of lower methylation capacity). Antioxidant capacity as indicated by plasma levels of cysteine and glutathione was significantly decreased in cases. Significant increase in odds ratio, allele frequency, and genotype distribution among autistic cases were found for RFC-1 80A>G, TCN2 776C>G, and COMT 472G>A genes. An increase in frequency of MTHFR 667C>T reached border line significance in autistic cases. Increased vulnerability to oxidative stress related to folate metabolism may contribute to development of autism.

Ramaekers et al. Belgium 2007 [62]	**Case control study** *Cases:* 25 children with early-onset low functioning autism *Controls:* 100 healthy age matched controls	Diagnosis established by DSM IV criteria and ADOS in conjunction with ADI around 3 years of age	Serum folate, cerebrospinal (CSF) folate, CSF 5MTHF folate receptor (FR) autoantibodies	There was no significant difference in serum folate levels between autistic cases and controls. The mean CSF level of MTHF was significantly lower than controls (27.3 nmol/L compared to 82.0 nmol/L). Autistic cases with low CSF MTFR had autoantibodies of blocking type against human FR. The reduced CSF folate in autistic cases was associated with FR autoantibodies blocking the folate binding site.

Schmidt et al. USA, 2011 [61]	**CHARGE population based case control study** *Cases:* 284 autistic and 141 children with ASD between ages 2–5 years *Controls:* 278 children with typical development	Diagnoses confirmed by: Autism Diagnostic Interview-Revised and Autism Diagnostic Observation Schedule Generic	Consumption of prenatal multivitamins, nutrient specific vitamins at any time during the period of 3 months before conception through pregnancy Maternal, paternal, and child samples were genotyped for MTHFR 667C>T MTHFR A1298C COMT 472G>A MTRR A66G TCN2	Use of prenatal vitamins during 3 months before and first month of pregnancy was associated with reduced risk of autism. (OR = 0.62, 95%. C.I. = 0.42–0.93). No association observed for vitamins intake during months 2 through 9 of pregnancy. Significant interactions were observed for autism between lack of perinatal maternal prenatal vitamin intake and both maternal MTFHR 667TT and CBSrs 234715GT+TT genotypes. With combined OR = 4.5, C.I. = 1.4–14.6 and 2.6 (1.2–5.4), respectively.

Schmidt et al. USA, 2012 [58]	**CHARGE case control study** *Cases:* 429 children with ASD *Controls:* 278 children with typical development. Cases and controls were frequency matched to age and catchment area distribution of autism cases with a 4 : 1 male-to-female ratio	The diagnoses of autism cases were confirmed by the Autism Diagnostic Interview-Revised (ADI-R) and by ADOS. The two initially identified subgroups, ASD and Autism, were later collapsed and results presented for combined ASD groups	Maternal folic acid intake was calculated from data on intake of multivitamins and prenatal vitamin including folic acid specific vitamins, cereal, and supplements during the index period (3 months before and throughout pregnancy) Maternal and cases MTHFR 667C>T was genotyped	The mean (±SEM) folic acid intake was significantly greater for typical development children than for mothers of children with ASD (*P* < 0.01) in first month of pregnancy. A mean folic acid intake of ≥600 µg versus <600 µg was associated with reduced risk of ASD (AOR = 0.62, 95% C.I. = 0.42–0.92) The association between folic acid and reduced ASD risk was strongest for mothers and children with MTHR 667C>T variant genotype. Periconceptional folic acid may reduce ASD risk in those with inefficient folate metabolism.

Suren et al. Norway, 2013 [53]	**Population based prospective study** The study sample of 85,175 children born in 2002–2008 was derived from Primary Norwegian Mother and Child Cohort study (MoBa), with the mean age of 6.4 years Exposure of interest was use of folic acid from 4 weeks before to 8 weeks after the start of pregnancy	Cases of ASD confirmed by linkages to the Norwegian Patient registry, capturing data for all children diagnosed with ASD by Norwegian health services	Specialist confirmed diagnoses of ASD	270 children who were in the study sample were diagnosed with ASD at the end of the study. In children whose mother took folic acid, 0.10% had ASD as compared to 0.21% in those unexposed to folic acid. The AOR for autistic children of folic acid users was 0.61, 95% C.I. = 0.41–0.90 as compared to folic acid nonusers. There was no association with Asperger syndrome or PDD-NOs, but power was limited. Use of prenatal folic acid supplements around the time of conception was associated with a significantly lower risk of ASD in the MoBa cohort.

*Enzymes and their alleles:

MTHFR: 5,10-methylenetetrahydrofolate reductase

COMT: catechol-O-methyltransferase

MTR: methyltetrahydrofolate homocysteine methyl transferase

TCN2: transcobalamin II

CBS: cystathionine *β*-synthase.

## Abbreviations

ASD: Autism spectrum disorder
PDD: Pervasive developmental disorder.
